# Circular RNAs are temporospatially regulated throughout development and ageing in the rat

**DOI:** 10.1038/s41598-019-38860-9

**Published:** 2019-02-22

**Authors:** E. Mahmoudi, M. J. Cairns

**Affiliations:** 1 School of Biomedical Sciences and Pharmacy, the University of Newcastle, Callaghan, NSW 2308 Australia; 2 Centre for Brain and Mental Health Research, University of Newcastle, Callaghan, NSW 2308 Australia; 3 Hunter Medical Research Institute, Newcastle, Australia

## Abstract

Circular RNAs (circRNAs) are covalently closed structural isoforms of linear mRNA which have been observed across a broad range of species and tissues. Here, we provide a comprehensive circRNAs expression catalogue for the rat including 8 organs of both sexes during 4 developmental stages using a public RNAseq dataset. These analyses revealed thousands of circular RNA species, many expressed in an organ-specific manner along with their host genes which were enriched with tissue-specific biological functions. A large number of circRNAs also displayed a developmental-dependent expression pattern and are accumulated during ageing. CircRNAs also displayed some sexually dimorphic expression, with gender associated differences observed in various tissues and developmental stages. These observations suggest that circRNAs are dynamically expressed in a spatial-, temporal- and gender-specific manner in mammals, and may have important biological function in differentiation, development and aging.

## Introduction

Circular RNAs (circRNAs), have been identified across a large number of species with a high abundance, stability and conservation^1–3^. CircRNAs are covalently closed single-stranded transcripts produced by joining a 5′ splice donor with an upstream 3′ splice acceptor of their linear parent molecules. Biogenesis of circRNAs is promoted by complementary sequences within the sites flanking circularized exons as well as specific alternative splicing factors such as Quaking (QKI) that binds to circRNA flanking sequences^4,5^. CircRNAs are widespread across the genome and mostly derived from protein-coding genes^6^. Their expression levels differ between tissues and does not necessarily correspond to the host genes^7^.

Although functional roles of circRNAs are yet to be well known, recent discoveries have shown that circRNAs are associated with gene regulation through various mechanisms. Interaction of circRNAs with transcription machinery and translation regulator proteins affects mRNA transcription and translation^8^. CircRNA can also regulate splicing of the linear isoform through specific interaction with the splicing factor, causing decreased level of the mRNA in favor of circRNA production^9^. However, the main function of circRNAs is perhaps inhibiting miRNA by specifically sponging them, resulting in a modified level of mRNA targets. The prominent example of this mechanism is CDR1as, which suppresses miR-7 function by acting as a miRNA sponge^6^. Loss of CDR1as in mouse brain caused deficient synaptic transmission via deregulation of miR-7 and miR-671 levels^10^. This observation was further confirmed by the ability of circHIPK3 to modulate cell growth by silencing multiple miRNAs such as miR-124^11^.

Recent evidence has shown that circRNAs are dynamically expressed in a specific spatial and temporal manner in mouse and human brain development^7,12^. The role of circRNAs in brain ageing was revealed in Drosophila where a global upregulation was observed in older animals^13^. This age-accumulation trend might be as a consequence of the circular structure giving these molecules increased stability compared to their mRNA counterparts^3,9^. Also, dynamic patterns of RNA splicing during ageing was reported in several organisms and tissues^14–18^, which could potentially impact the abundance of circRNA. CircRNAs analysis in mice suggested there was a global increase in levels of circRNA in aged brain (22-month-old) tissues compared to young mice (1-month-old), whereas no alteration was detected in the heart^19^.

The complete expression profiles of circRNAs across a mammalian body and their regulation during development is still unknown. Here we map circRNA expression across in 8 tissues during 4 developmental stages for both genders in rat using a public total RNA-Seq dataset^20,21^ (n = 240). This data shows that circRNAs are present throughout the rat body but are at relatively higher levels in the brain. They are mostly derived from protein-coding regions with an expression trend that usually diverged from their parental linear isoforms. CircRNAs overall were upregulated during organ development and aging, highlighting a developmentally-dependent expression pattern. We detected many circRNAs that were tissue-specific with biological functions related to their parental mRNA function. They also have potential to sponge away the activity of developmentally significant miRNA and form complex regulatory networks reflected in mRNA expression levels. Analysis of circRNAs expression in male and female suggested there is sexual dimorphism related to these molecules in all tissues and developmental stages.

## Results

### CircRNAs are expressed in all tissues and enriched in the brain

**Figure 1:**
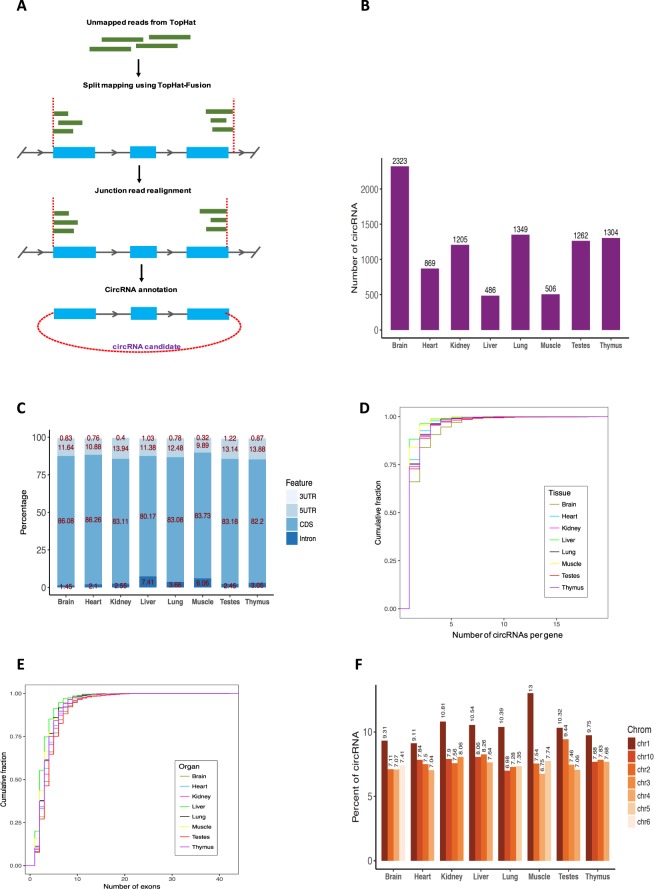
Comprehensive landscape of circRNAs in rat. (**A**) Schematic illustration of the computational pipeline used to identify circRNAs. (**B**) Number of circRNAs detected in rat categorized by organ. (**C**) classification of the discovered circRNAs by genomic features. (**D**) Distributions of gene numbers for circRNAs in each organ. (**E**) Distributions of exon numbers for circRNAs in each organ. (**F**) Top 4 chromosomes with the largest number of circRNAs in each organ.

A comprehensive circRNA expression catalog of the rat was generated using a deep-total RNA sequencing dataset accessible at NCBI GEO database^20^ (GEO GSE53960), including segmented reads from 8 organs across four developmental stages for both males and females. The tissue profiles include brain, heart, lung, liver, kidney, muscle, testes and thymus, for developmental time points; juvenile (2-weeks old), adolescence (6-weeks old), adult (21-weeks old) and aged (104-weeks old). For each developmental stage, 8 rats (4 female and 4 male rats) were evaluated with 4 replicates each. CircRNA candidates were identified using the CIRCexplorer algorithm (Fig. 1A) with an extra step where reads with no back-splicing were filtered out in each sample to improve the detection quality. In total, we detected 5,058 distinct circRNA candidates, with a minimum of two reads per sample spliced from 2,578 genes (Supplementary Table 1). CircRNA expression analysis clearly demonstrated higher abundance and diversity in the brain, followed by lung, thymus, testes and kidney (Fig. 1B). The higher expression level of circRNA in brain is consistent with findings from human and mouse^7,22^. The vast majority of circRNAs (>99%) in each organ were produced from annotated protein-coding genes. CircRNAs distributed across various genomic regions including 5′ UTR, 3′ UTR, intron, but most commonly from coding exons, CDS, where over 80% of circRNAs originated (Fig. 1C). There was a preference for circRNA parental genes that produce a single circRNA (>65%), followed by genes generating 2 and 3 circRNAs (Fig. 1D). Overall, we observed a broad diversity in the number of exons embedded within circRNAs, with a large proportion containing 2–4 exons (>55%). The exceptions were liver and muscle tissues which had the least exons, with the majority having 1–3 exons per circRNA (Fig. 1E). While circRNAs were distributed across the genome, 4 chromosomes including chr1, 2, 4, and 10 produced the highest number of circRNAs with respect to all organs (Fig. 1F).

### circRNAs are differentially expressed during development

**Figure 2:**
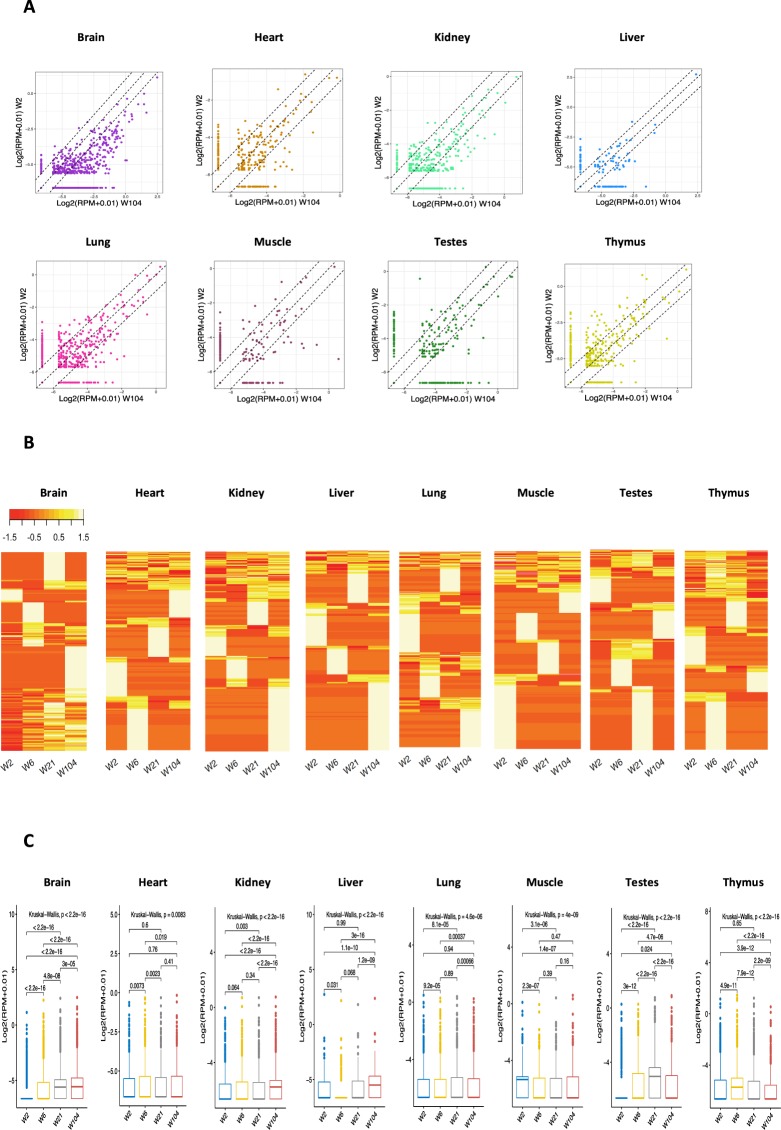
Analysis of circRNAs expression across development. (**A**) Scatter plot of circRNAs fold changes between 2- week old and 104-week old rats. (**B**) Heatmap comparison of circRNAs expression across 4 developmental time points. The abundance is depicted on a scale from red (low expression) to yellow (high expression). (**C**) Average expression of circRNA at 4 developmental stages calculated in RPM values (reads per million mapped reads) and ANOVA test for all the stages.

We examined the global expression changes of circRNAs during 4 developmental stages as described above. The results showed differential expression of circRNA across development in different organs (Fig. 2). Overall, circRNA levels increased throughout the life span of the rats in the brain and kidney, with juvenile rats producing the lowest circRNA levels followed by an ascending trend during development (Fig. 2C). Liver and lung also displayed the highest circRNA expression in the last stage of development whereas the highest abundance in testes was shown in the second stage. Quite the opposite was observed in thymus, with circRNAs abundance tending to decrease, from adolescence, as the animal matures reaching its minimum level at old age (Fig. 2A–C). The least global alteration was observed for heart and muscle in response to ageing where only 3 pairwise comparisons were significant, with the highest average of circRNA observed in stage 2 and 1 respectively (Fig. 2C).

**Figure 3:**
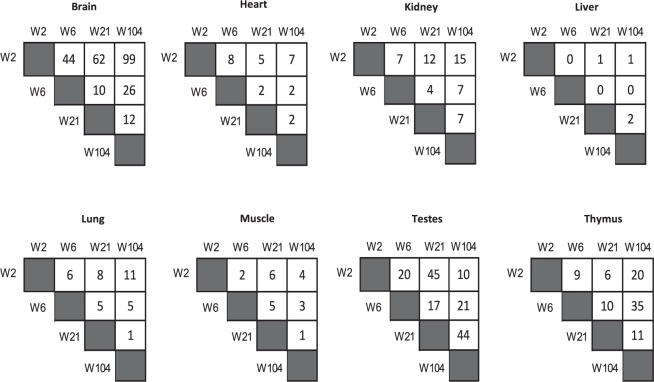
Development-dependent expression of circRNAs in rat organs. (**A**) Differential Expression analysis of circRNA between 4 developmental time points from 2-week old to 104-week old for 8 organs based (Wilcoxon test, P-value < 0.05 and FC >2).

In order to identify the individual circRNAs with statistically significant alterations during rat aging, we performed a time course differential gene expression analysis by comparing any two proximal developmental stages using thresholds of Wilcoxon test P-value < 0.05 and Fold Change >2. As shown in Fig. 3, there is a significant change in the expression across life span in the studied organs. Overall, we detected 640 circRNAs differentially expressed during development in 8 organs (Fig. 3). The brain was observed the have the greatest number of developmentally regulated circRNA (253) with the largest change occurring in 2-week compared to the 104-week time point. This was followed by testes, thymus and kidney representing 157, 91 and 52 developmentally altered circRNAs, respectively (Fig. 3). However, liver had the lowest differential expression change with only 4 circRNAs. Looking at the pairwise comparison results for all organs, it is notable that the most differential alteration was found for 2-week versus 104-week and 2-week versus 21-week time points.

### CircRNAs have organ-dependent expression pattern

**Figure 4:**
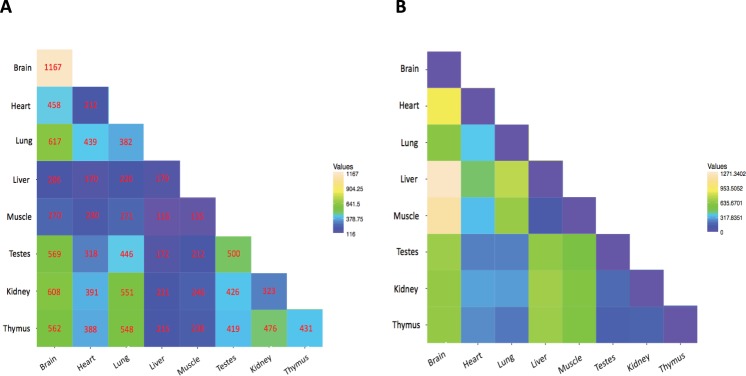
Pairwise comparison of circRNAs between different organs. (**A**) Number of circRNAs overlapped between any two rat organs. (**B**) Euclidean distance for circRNAs based on number of shared circRNAs between different organs.

To identify organ enriched circRNAs, we compared the expression level in each tissue respectively to that from the other 7 organs using and RPM expression cutoff value of 0.05 in at least one sample in each organ. In total 3,329 circRNAs were found to have organ-specific expression, as detailed in Fig. 4 and Supplementary Table 2. Brain ranked first with 1167 (50%) specific circRNAs and then testes and thymus represented the highest number of tissue- specific circRNAs with 500 (40%) and 431 (36%) circRNAs, respectively. The least specificity was found for heart, liver and muscle (Fig. 4A), possibly relating to a lower number of detected circRNA in these organs. We determined the distance matrix between any two of organs using Euclidian method to visualize the similarity of organs in terms of circRNA profile. As shown in Fig. 4B, the most similarity was observed for liver-muscle (distance = 131.8), thymus-testes (164.5) and thymus-kidney (166.4) and conversely, brain-liver (1271.3), brain-muscle (1168.8) and brain-heart (859.15) represented as the most distantly related organs in respect to circRNA expression.

Interestingly, only 72 circRNAs were constitutively expressed in all organs throughout development (Supplementary Fig. 1). Despite this similarity, the abundance of these molecules was higher in brain, kidney, lung and thymus (Supplementary Fig. 1). Their prevalence in all tissues and stages suggest these circRNAs may have some basic function in cell biology as shown for their host genes in Supplementary Table 3.

### Tissue-specific circRNAs related to the biological functions of their organ

**Figure 5:**
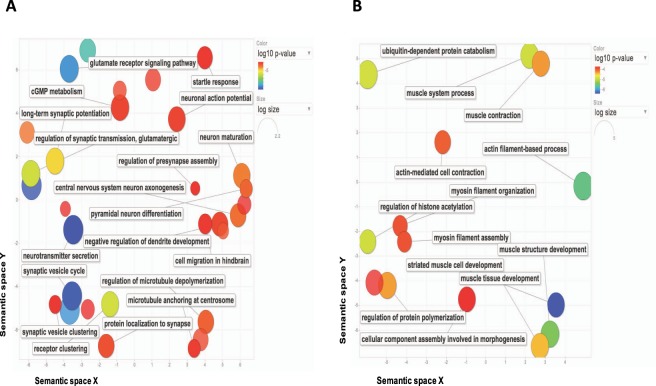
Gene Ontology enrichment analysis of circRNAs host genes. (**A**) The scatter plot of GO clusters significantly enriched in brain. (**B**) The scatter plot of GO clusters significantly enriched in heart. The scatter plot indicates the clusters (after redundancy filtration) in a two-dimensional space by applying multi-dimensional scaling to a matrix of GO terms semantic similarities. The color of bubble shows corrected P-value and the size of circle represents the frequency of the GO term in the GOA database.

To gather insight into the possible function of the organ-specific circRNAs, we performed Gene Ontology analysis of their respective host genes in each organ. Generally, there was significant enrichment (FDR <0.05) of clusters specifically related to the tissues biological function that was related to its host organ. Supplementary Table 4 includes a list of all the organ-enriched GO groups. For instance, brain circRNA host genes were enriched in neurotransmitter secretion, synaptic activities and neuron maturation (Fig. 5A), while in heart we observed enrichment of cardiac muscle differentiation and development, muscle contraction and myosin filament organization (Fig. 5B). Other examples of organ-specific enrichment include urea cycle in liver, sperm mortality in testes, T cell proliferation in thymus, regulation of cell-matrix adhesion in lung, striated muscle myosin thick filament assembly in muscle, and organonitrogen compound catabolic process in kidney (Supplementary Table 4).

### Differential expression of circRNA was not associated with change in host gene mRNA

**Figure 6:**
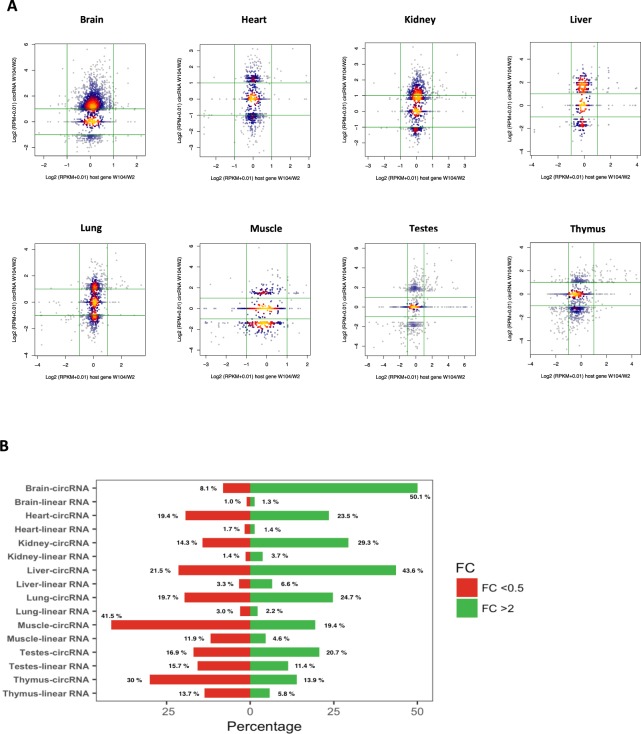
CircRNA and mRNA expression changes. (**A**) Comparisons of circRNAs expression change between initial and final developmental stages to the mRNA counterparts at the same developmental stages. (**B**) Barplots comparing the percentage of circRNAs differentially expressed (FC >2) between 2-week and 104-week old rats to the mRNA counterparts in each organ. Green bars represent upregulation and red indicated downregulation.

To examine whether differential circRNA expression was related to changes in their linear hosts, we compared corresponding transcripts in all organs during development (Fig. 6). Surprisingly, circRNAs were observed to have greater differential expression across development compared to their linear counterparts in all the tissues analysed. For example, in brain, there was a substantial alteration in circRNAs (over 58%) during development, whereas, only 2.3% of their linear isoforms showed an expression change (Fig. 6B). Other organs represented a similarly large difference in expression profiles of circRNAs and host transcripts, except for the testes, where the differential expression profiles were more similar at 37.6% for circRNAs and 27.1% for the linear isoforms (Fig. 6B). In order to explore the correlation between the abundance of circRNA and their linear transcripts we also performed correlation which suggested there is a small positive relationship between the two spliceoforms classes at different stages (Supplementary Table 6). These observations suggest that while the circRNAs response to development is more dynamic, their transcription and canonical splicing is related to their linear counterparts.

### Sexual dimorphism observed in circRNAs expression

**Figure 7:**
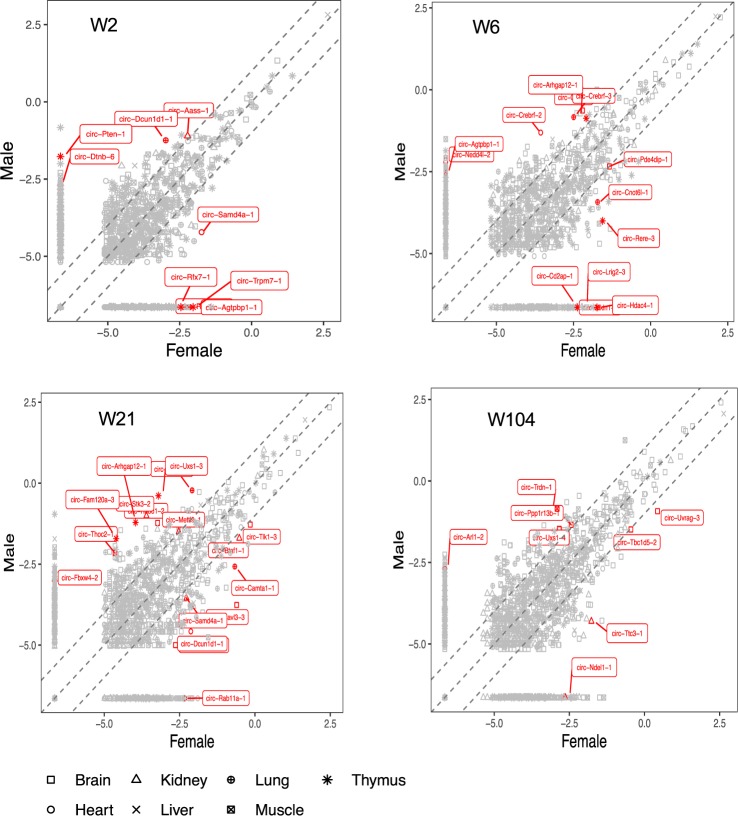
Sex differences of circRNA pattern in rat. CircRNAs expression (log2 RPM values) for both genders are plotted using pooled data of seven organs in each developmental stage. The red points with circRAN names are significantly expressed circRNAs between male and female.

We determined gene expression profiles for matched tissues between male and female rats at all developmental time points and observed 47 circRNAs to be differentially expressed (Wilcoxon test P-value < 0.05, FC >2) (Fig. 7, Supplementary Table 5). Different patterns were observed in 6 organs particularly in brain, thymus and lung with 15, 11 and 9 circRNAs displaying gender-specific expression in comparison with other tissues. Liver was found the only organ that was relatively devoid of sexual dimorphism. It was notable that most differentially expressed circRNAs were found at 21 weeks, in adults (Supplementary Table 5). We looked if there is a sex dominance at any age stage and found no significant dominance at any developmental time point.

### Age-accumulated circRNAs may regulate target mRNAs across development

**Figure 8:**
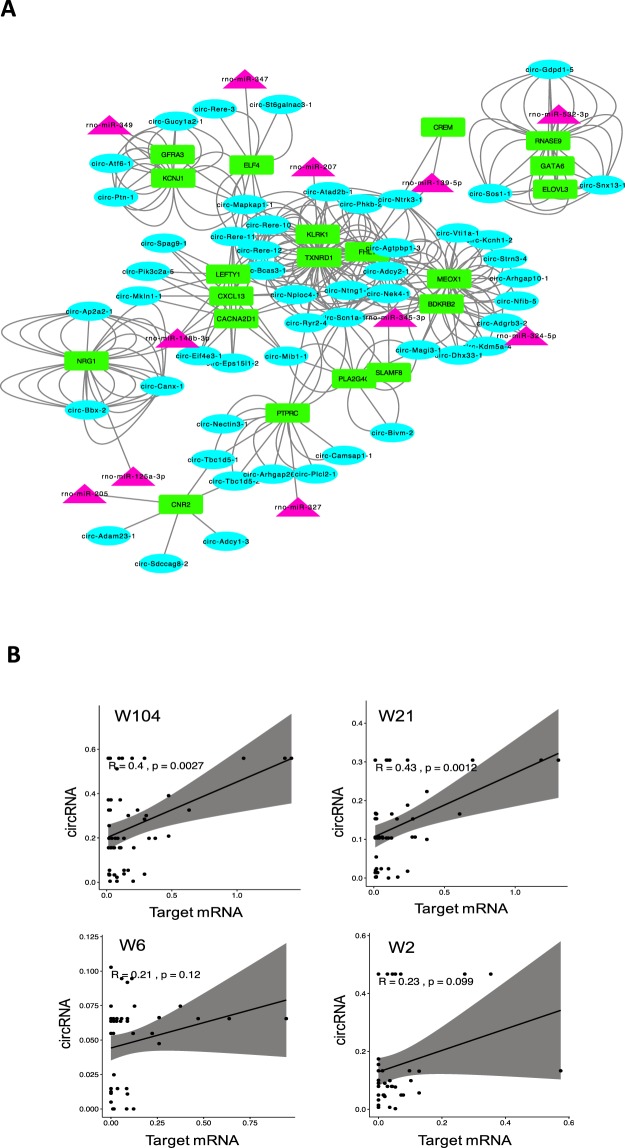
Prediction of interaction network of circRNA-miRNA-mRNA. (**A**) Interaction network of age-accumulated circRNAs with developmentally increased mRNAs via targeting miRNAs. Purple Circles represent circRNA, pink triangles miRNA and green rectangles mRNA. (**B**) Correlation between the expression levels of circRNA and mRNA targets at 4 different developmental stages.

Given the accumulation of circRNAs expression during development (Fig. 2) we suspect this may have functional significance and could contribute to the pathophysiology of ageing. A key function of circRNA is to sequester miRNA by sponging, resulting in alteration of mRNA targets^6,10^. To investigate the function of the ageing-associated circRNAs in the brain (n = 246), as the most age-associated tissue, we predicted the miRNAs that could potentially bind to these circRNAs using TargetScan^23,24^ and miRanda^25^ algorithms. A total of 141 interactions were identified, where 99 circRNAs associated with 28 miRNAs. Next, we determined the potential mRNA targets of the interacting miRNAs and matched them to the developmentally increased mRNAs (Fold change ≥ 1.5) extracted from the sequencing data. We found 20 distinct mRNAs were targeted by the some of these miRNAs, as illustrated in the interaction network constructed for the associating circRNAs, miRNAs and mRNAs (Fig. 8A). The correlation between the age-accumulated circRNAs and the mRNA targets was calculated as shown in Fig. 8B. These interactions suggest that the age-associated circRNAs may have an impact on ageing processes by modulating mRNAs expression through sponging miRNAs and decreasing their gene silencing activity.

## Discussion

In this study, we systematically analyzed circRNAs expression in rat to provide a circRNA body map across 8 organs at 4 developmental time points for both male and female using a rRNA depleted total RNA-Seq dataset including 240 samples^20^. In total, 5,056 circRNA candidates were detected, each with at least two reads per sample, which were hosted by 2,578 genes. In accordance with other reports^3,7,26^, multiple circRNAs with different back-splice junctions were produced from the same gene loci in mostly protein-coding regions (Fig. 1).

We found that many circRNAs were enriched in the tissues from particular organs (Fig. 4), suggesting they display a temporospatially restricted expression pattern. CircRNAs were generally in higher abundance in the brain compared to other tissues (Fig. 1B) which is consistent with previous observations in other organisms^6,7,22^. This also accorded with observations in the mouse, where circRNAs expression in the brain were shown to be significantly different to the heart^19^. While this patterning may be associated with the tissues-specific biological activities, it was also consistent to some extent with host gene expression as many were significantly associated with organ related biological processes in gene set enrichment analysis (Supplementary Table 4).

We also present evidence that circRNAs are dynamically regulated across development in all of the tissues analysed (Fig. 3A,B), with a large proportion of circRNAs (89.8%) showing an alteration at least in one stage with only a small proportion of molecules remaining unchanged throughout development. Profiling circRNAs in multiple brain tissues at six time-points of fetal porcine development revealed differential expression of circRNAs during development, suggesting a temporal regulation of circRNAs^12^. Another study of circRNAs in development identified a group of circRNAs to be differentially regulated at different developmental stages, E18 to P30, in the mouse hippocampus^22^. A temporal expression pattern was also observed for circRNAs during neuron differentiation and maturation at different time points^7^. These findings suggest that circRNAs are involved in defining cell/tissue identity and that circularization is likely important for cell/organ maturation, as many of these molecules are regulated at different times during development. The observed fluctuation in expression of many circRNAs at different stages of development in our data demonstrate that each circRNA may have both positive and negative impact across development. We also observed an overall accumulation of circRNAs during development (Fig. 2). Notably, the upregulation was larger and more constant in brain where circRNAs abundance increased by more than three times in aged as compared to juvenile rats. A bias for circRNAs accumulation was also reported in ageing cortex and hippocampus of mouse^19^. More evidence of circRNA increase in the ageing CNS was observed in Drosophila, with a predominant accumulation of circular RNAs found in the brain compared to any tissue^13^. Similarly, circRNAs showed a global upregulation between embryonic day 18 and 1 month of age in mouse brain embryonic day 18 and 1 month of age^22^. This expression feature of circRNAs suggest that they could serve as biomarkers of development and ageing, particularly when compared to linear RNAs, which show comparatively smaller changes^27,28^. In support of these previous observations, our analysis showed that linear transcripts hosting circRNAs were not subject to the same level of change during development (Fig. 6A,B). While the high stability of circRNAs may be one of the drivers of this change, it is possible that back splicing may also increase with age. Therefore, we compared expression of two genes, QKI and ADAR1, previously known as circRNA biogenesis regulators^5,7^ with circRNA levels during development to examine whether the observed circRNA alterations is caused at the biogenesis stage. Our results however, did not support this hypothesis with no correlation between circRNA abundance and known circRNA biogenesis genes, suggesting that there might be other genes contributing to circRNA formation or that regulation of the steady state levels of these transcript isoforms may occur at the posttranscriptional level. The observed increase in circRNA levels in ageing also suggest that these molecules might associate with age-related processes and diseases.

Given the temporospatial specificity of circRNA throughout mammalian development it seems highly likely that they have genomic regulatory functions. It has been suggested that these molecules can modulate their host genes by competing for transacting proteins and miRNA that may be suppressing activity of the coding transcript. There is also the suggestion that some of these molecules may interact directly with genes and modify their transcription. A small number of these have even been shown to be amenable to ribosome entry and allow their sequence to be translated into protein. Perhaps the most substantial regulatory function established for circRNA is miRNA regulation^8^ and there is now compelling evidence to support their function as competing endogenous RNA (ceRNA). This means they essentially work by sponging miRNA and reducing their capacity to direct gene silencing^6,10^. We investigated age-accumulated circRNAs interaction with miRNA and their mRNAs targets by generating a network of circRNAs-miRNA-mRNA interactions for brain. The results indicated that many miRNA could be sponged by the age-associated circRNAs, resulting in a mRNA increase during brain development (Fig. 7). MiRNA are differentially expressed during ageing in a tissue-specific manner in both humans and rodents^29–32^, suggesting that these molecules modify target genes that are active in ageing-related pathways^32^. Ageing was also linked to a substantial alteration in gene expression in various tissues of mammals and human^14,33–35^. Comprehensive analysis of transcriptomic activities indicated a differential expression of mRNA across the life cycle of the rat in various organs^21^. These findings suggest that the circRNA accumulation during ageing may influence mRNA associated with the pathophysiology of ageing, by the suppression of the gene silencing activity of miRNA.

Finally, we analyzed circRNAs expression in males and females for all developmental stages and found a number of circRNAs demonstrated a gender-specific expression pattern (Fig. 7). Part of this bias may be due to circRNAs derived from sex chromosomes. It is also possible that gender-specific physiological conditions and different environmental factors such as sex hormones might play a role in the observed expression bias. Furthermore, there is evidence of sex differences in organ structure and development and function^36–38^. This has been associated with differential gene expression patterns between male and female^39^. Therefore, circRNAs profiles in both genders are likely to be affected by organ changes resulted from sex differences.

During review of our study another group reported a positive correlation between the circRNA and host genes expression in the same data resource^40^. This was broadly in agreement with our interpretation, particularly between the circular splicing and highly expressed genes. They also found a higher tissue specificity for circRNAs compared to their linear isoforms and significant changes during development, with the greatest alteration found in brain and testes. In the current study we extend further to provided differential expression analysis of individual circRNAs across aging for all the sequenced organs. We also report the sexual dimorphism of circRNA expression and explored potential function of circRNAs by performing in silico analysis of target interactions and consequences.

In both studies circRNA expression was observed in total RNA sequencing read-count data. As this includes linear transcript reads, the efficiency of circRNA identification could be improved by depletion of linear RNAs using treatment with RNase R as described by the methodology known as CircleSeq^41^. As circRNAs have much lower abundance compared to mRNA there is substantial competition with linear RNA fragments during total RNA sequencing library construction which can be reduced by linear RNA depletion. In some cases circRNA detection in total RNAseq reads can be compromised when linear spliced and back-spliced reads cannot be differentiated due to sequence similarity, potentially resulting in false positive detection of circRNA. While CircleSeq does provide higher sensitivity and specificity for circRNA analysis, it is more difficult to ascertain the relationship to host transcript expression and functional relationship to putative target genes mediated by miRNA sponging. This read-count data is also more difficult to normalize and therefore may be less quantitative.

In summary, this analysis provides a comprehensive anatomical map of circRNAs expression in the rat from juvenile to old age in both genders. We indicated that circRNAs are regulated in an organ-, development and gender- specific manner in these animals, which can serve as a resource for future research addressing the function of these circRNAs in mammals.

## Methods

### RNA sequencing data

Ribosomal RNA depleted total RNA sequencing data from NCBI Gene Expression Omnibus (GEO) database^20^ with accession GSE53960 was downloaded^21^. The raw data were then converted into the Fastq format using sratoolkit. The data included 240 samples for 8 organs of both female and male across four developmental stages. The organs included brain, heart, lung, liver, kidney, muscle, testes and thymus, and the developmental time points were juvenile (2-weeks old), adolescence (6-weeks old), adult (21-weeks old) and aged (104-weeks old). For each developmental stage 8 rats including 4 female and 4 males rats with 4 replicates each. The original study was conducted in accordance ethical and scientific approval from the National Center for Toxicological Research Institutional Animal Care and Use Committee, with animals housed and euthanized according to the NIH guidelines^21^.

### Computational pipeline for circRNAs prediction

CIRCexplorer^42^ was used to predict back-splice junction candidates (Fig. 1A). According to the pipeline, sequence reads were initially mapped with TopHat 2.0.9 (parameters: -a 6–microexon-search -m 2) to rat reference genome rn4. Unmapped reads were then collected and mapped onto the reference genome using TopHat-Fusion (parameters:–fusion-search–keep-fasta-order–bowtie1–no-coverage-search). All the reads that were mapped on the same chromosome, however, in a reverse order were extracted as candidate back-spliced junctions. To identify the positions of the acceptor or donor sites of each back-spliced event, the back-spliced junction reads were remapped against the gene annotations. Using a custom script, all the junction reads with an alignment shift against canonical splice sites were corrected and then reads mapped on non-canonical splice sites or different genes were discarded. In the final step, all the back-splice candidates were checked for supporting reads and those with more than one read were regarded as circRNAs.

### CircRNAs expression analysis

To estimate the expression of circRNAs we quantified the number of reads spanning back-spliced junctions (circular RNA reads). We then normalized the back-spliced junction read counts by sequencing depth in each sample. As such, circRNA reads were divided by the total number of mapped reads in each sample to obtain RPM (mapped back-splice junction Reads Per Million mapped reads) values^42^. The relative expression of circRNAs were determined by comparing RPM values between samples.

### mRNA expression analysis

For mRNA expression analysis, reads were aligned to the rat reference genome rn4 using TopHat 2.0.9 (parameters: -a 6–microexon-search -m 2) and mapped reads were collected. Aligned reads were then fed into cufflinks (v2.2.1) and RPKM values were obtained for each gene. To avoid infinite values, we added a value of 0.01 to the RPKM value of each gene before log2 transformation.

### Functional enrichment analysis

To determine the biological processes of the organ-specific circRNAs and also circRNAs shared across all organs, Gene Ontology enrichment analysis was carried out for the host genes of these circRNAs using GO (http://geneontology.org)^43^. A P-value of 0.05 and FDR <0.05 was set to identify significant GO clusters. The enrichment analysis graphs were created using REVIGO^44^.

### MiRNA binding site prediction and network construction

We determined age-accumulated circRNAs by selecting those with an increased expression across development. For age-upregulated mRNAs, we selected mRNA that were differentially expressed during brain development (Fold change ≥ 1.5). To detect miRNA binding sites within circRNAs, exon sequence of the circRNAs were extracted using rn4 annotation. MiRNA binding sites within the circRNAs and mRNAs were detected using TargetScan_60 (http://www.targetscan.org/vert_71/)^23,24^ and miRanda (http://www.microrna.org/microrna/home.do)^25^ algorithms. We applied a context score threshold cutoff <−0.2 for TargetScan, and scores >140 and DG <10 kcal/mol for miRanda to obtain reliable interactions. The circRNAs-miRNA-mRNA network were constructed using Cytoscape tool v.3.5.1 (http://www.cytoscape.org/)^45^.

## Supplementary information

Supplementary Figure 1

Supplementary Table 1

Supplementary Table 2

Supplementary Table 3

Supplementary Table 4
