# Supplementary material for: Circular RNAs are temporospatially regulated throughout development and ageing in the rat
Source: Sci Rep. 2019 Feb 22;9:2564. doi: 10.1038/s41598-019-38860-9 (PMC6385508; doi:10.1038/s41598-019-38860-9)
Supplement: Supplementary file 1 — Supplementary Figure 1 [file 41598_2019_38860_MOESM1_ESM.pdf]

Circular RNAs are temporospatially regulated throughout development and ageing in the rat

Mahmoudi E, Cairns MJ

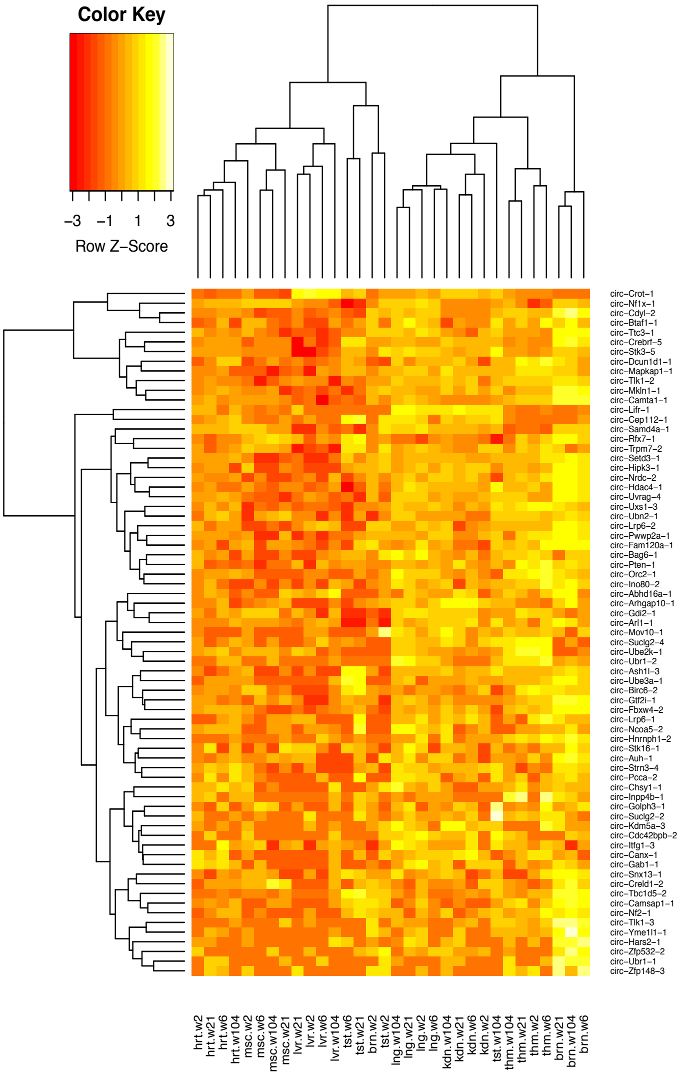

Supplementary Figure 1. Hierarchical and heatmap clustering of the shared circRNAs at different developmental stages.

Organ	Week 2		Week 6		Week 21		Week 104	
	Female*	Male	Female	Male	Female	Male	Female	Male
Brain	1	0	2	2	2	3	2	2
Heart	1	1	1	0	0	1	0	0
Kidney	1	0	0	0	3	2	0	2
Liver	0	0	0	0	0	0	0	0
Lung	1	0	2	1	1	2	1	0
Muscle	1	0	0	0	0	0	0	1
Thymus	1	2	1	4	3	0	0	0

Supplementary Table 5. Number of circRNAs that were female-or male-dominant.
*Female: number of female-dominant circRNAs, DE circNRA between female and male: FC > 2 (Female/Male) with *p*-value <0.05. Male-dominant circNRA were detected as follows: FC > 2 (Female/Male) with *p*-value < 0.05.

Organ	Week 2		Week 6		Week 21		Week 104	
	R coefficient	P-value	R coefficient	P-value	R coefficient	P-value	R coefficient	P-value
Brain	0.02	0.33	0.091	0.000	0.064	0.0019	0.11	0.000
Heart	0.02	0.33	0.11	0.001	0.011	0.76	0.41	0.23
Kidney	0.01	0.67	0.11	0.00	0.83	0.00	0.065	0.024
Liver	0.09	0.03	0.02	0.58	0.16	0.00	0.02	0.6
Lung	0.06	0.01	0.06	0.01	0.06	0.01	0.05	0.06
Muscle	0.05	0.22	0.15	0.00	0.11	0.01	0.15	0.00
Testes	0.18	0.00	0.15	0.00	0.28	0.00	0.25	0.00
Thymus	0.13	0.00	0.16	0.00	0.06	0.01	0.04	0.1

Supplementary Table 6. Correlations between circRNAs and their host genes in each stage.
